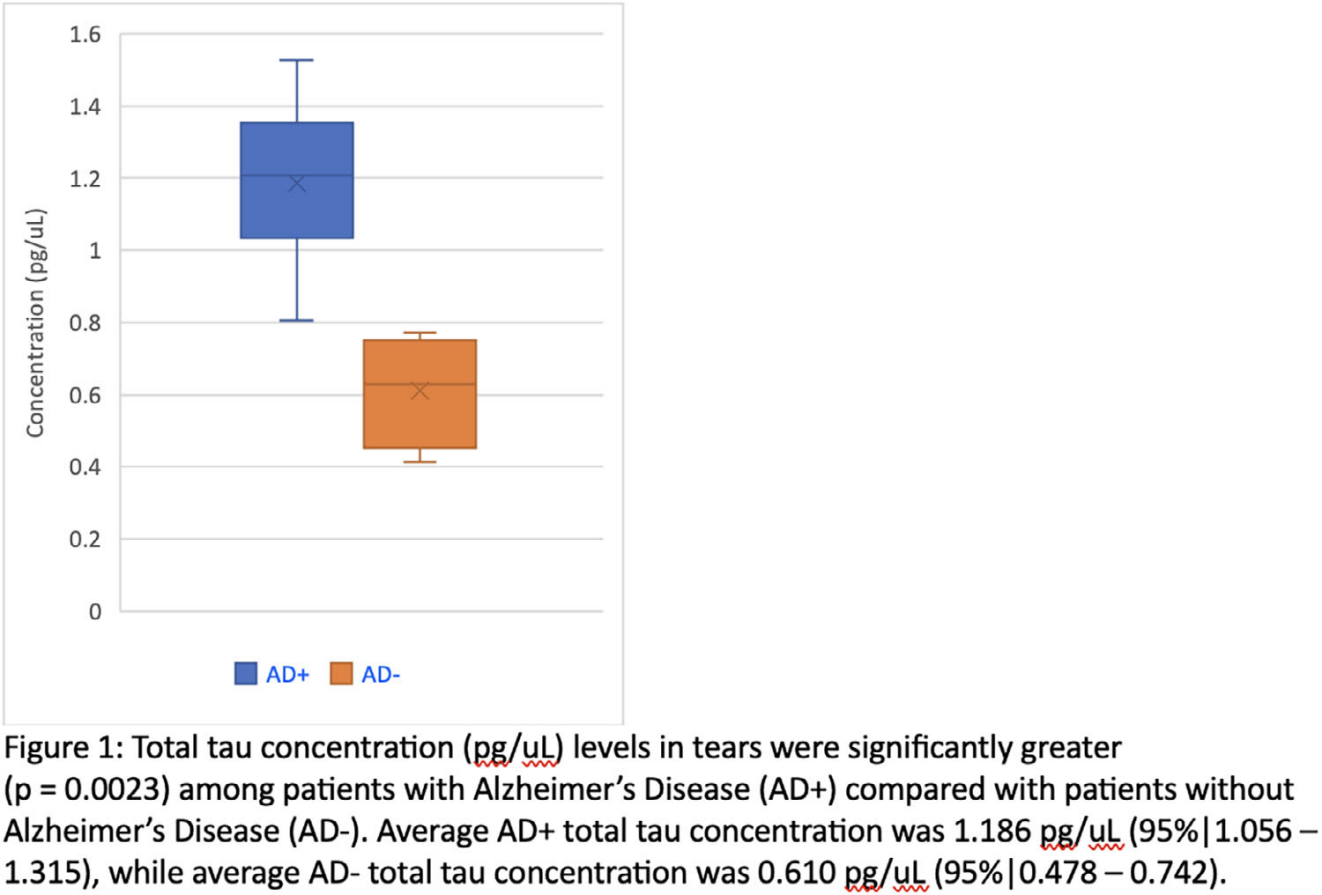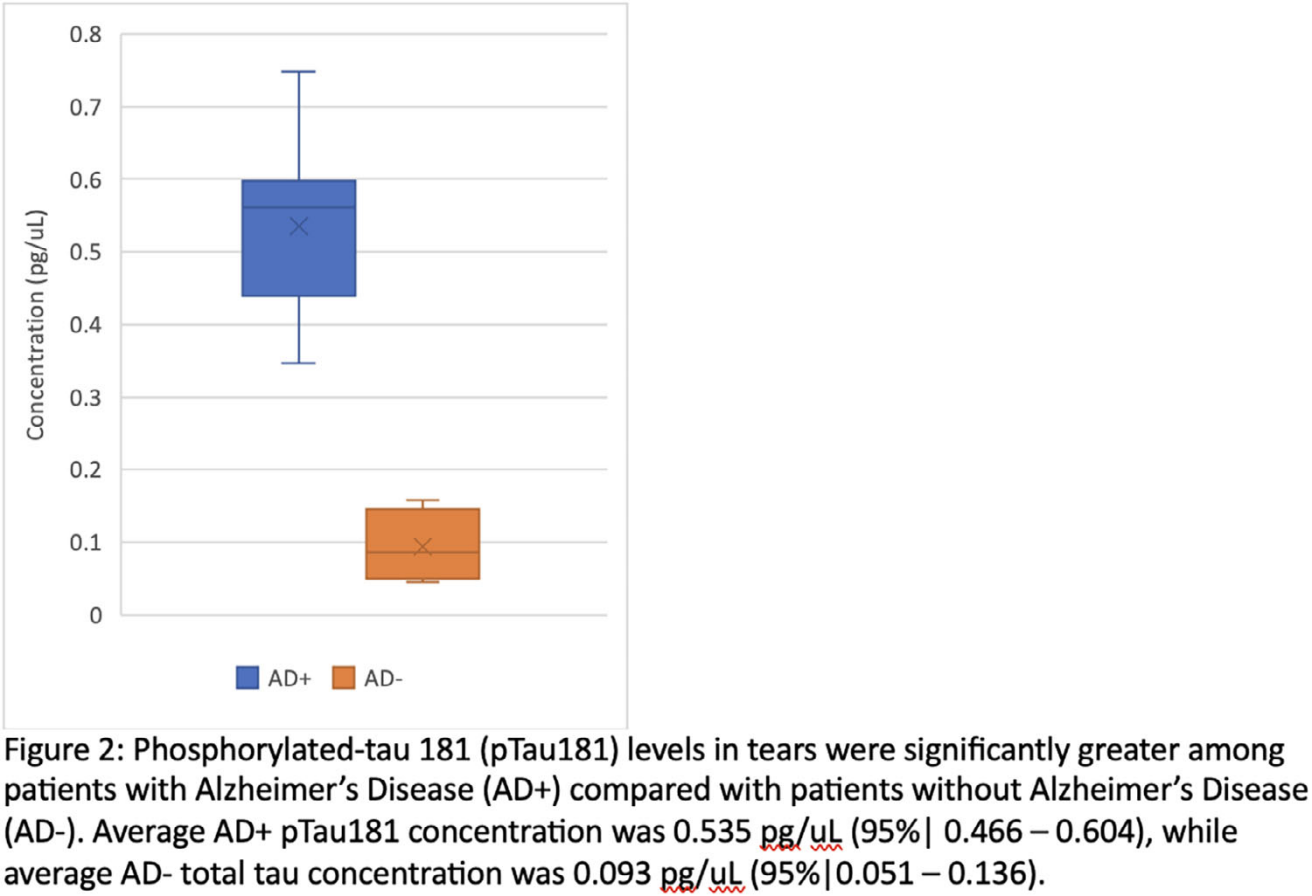# Tear Biomarkers as Indicators of Alzheimer's Disease

**DOI:** 10.1002/alz.089809

**Published:** 2025-01-09

**Authors:** Anshule Takyar, Maisie Bailey, Richard Trantow, Danilo A Sanchez Coronel, Hector Hugo Garcia, Robert H Gilman, Monica M Diaz

**Affiliations:** ^1^ Johns Hopkins School of Public Health, Baltimore, MD USA; ^2^ Oregon Health Sciences University School of Medicine, Portland, OR USA; ^3^ Alaska Department of Health, Anchorage, AK USA; ^4^ Instituto Nacional de Ciencias Neurologicas, Lima Peru; ^5^ Universidad Peruana Cayetano Heredia, Lima, Lima Peru; ^6^ University of North Carolina at Chapel Hill School of Medicine, Chapel Hill, NC USA

## Abstract

**Background:**

The prevalence of Alzheimer’s disease (AD) is increasing worldwide, particularly in low‐ to middle‐income countries (LMICs). Resource limitations and time constraints in many LMICs make AD screening and diagnosis difficult in the clinical setting. Neurodegenerative biomarkers in human tears may be associated with neurodegenerative diseases, but its potential has yet to be investigated in AD. Amyloid‐beta, tau, α‐synuclein, and neurofilament light (NfL) levels in tears were compared in patients with AD (AD+) and age‐matched negative controls (AD‐).

**Methods:**

Samples were collected from a cohort of AD+ (N=26) and age‐matched AD‐ (N=9) individuals at a public neurological hospital in Lima, Peru. Tears were collected on Schirmer strips, which were dried and frozen. Proteins were then extracted into a PBS‐based buffer and tested for biomarkers using commercial ELISA kits, Luminex assays, and digital ELISA methods from Quanterix and NanoMosaic. Biomarker burdens were compared between AD+ and AD‐ patients using digital ELISA results. Amyloid‐beta 40, Amyloid‐beta 42, total tau, phosphorylated‐tau181 (pTau181), α‐synuclein, and NfL burdens were evaluated using various ELISA methods, and Human TIMP‐1 was used as a control.

**Results:**

Initial analyses using commercial ELISA and Luminex methods proved inconclusive. NfL burden was evaluated using the Quanterix digital ELISA platform showed a detectable protein burden in 3 AD‐ samples and 1 AD+ samples, but remained statistically insignificant (p = 0.45). A custom digital ELISA assay on the NanoMosaic platform showed AD+ status was associated with elevated Total Tau (p = 0.0023) and pTau181 (p = 0.0016) burden in tear proteome as compared to AD‐ samples, shown in Figures 1 and 2.

**Conclusions:**

Due to considerably lower limits of detection, the Quanterix and custom NanoMosaic assays allowed for increased sensitivity of biomarker detection in human tears. Our results indicate a significant association between AD+ status and elevated Total Tau and pTau181 levels in tears, and suggest the potential for tears to be pursued as a potential non‐invasive and accessible addition to AD screening and diagnosis, particularly for LMICs.